# Diacylglycerol kinase ζ interacts with sphingomyelin synthase 1 and sphingomyelin synthase‐related protein via different regions

**DOI:** 10.1002/2211-5463.13628

**Published:** 2023-05-21

**Authors:** Masataka Furuta, Chiaki Murakami, Yuki Numagami, Rika Suzuki, Fumio Sakane

**Affiliations:** ^1^ Department of Chemistry, Graduate School of Science Chiba University Japan; ^2^ Institute for Advanced Academic Research Chiba University Japan

**Keywords:** diacylglycerol kinase, sphingomyelin synthase, sphingomyelin synthase‐related protein, sterile α motif domain

## Abstract

We previously reported that diacylglycerol (DG) kinase (DGK) δ interacts with DG‐generating sphingomyelin synthase (SMS)‐related protein (SMSr), but not SMS1 or SMS2, via their sterile α motif domains (SAMDs). However, it remains unclear whether other DGK isozymes interact with SMSs. Here, we found that DGKζ, which does not contain SAMD, interacts with SMSr and SMS1, but not SMS2. Deletion mutant analyses demonstrated that SAMD in the N‐terminal cytosolic region of SMSr binds to the N‐terminal half catalytic domain of DGKζ. However, the C‐terminal cytosolic region of SMS1 interacts with the catalytic domain of DGKζ. Taken together, these results indicate that DGKζ associates with SMSr and SMS1 in different manners and suggest that they compose new DG signaling pathways.

## Graphical abstract

The sterile α motif domain (SAMD) of sphingomyelin synthase (SMS)‐related protein (SMSr) binds to the C‐terminal half catalytic domain of diacylglycerol kinase (DGK) ζ. Moreover, the C‐terminal cytosolic region of SMS1 interacts with the catalytic domain of DGKζ. SMSr‐SAMD associates with DGKδ‐SAMD and activates DGKδ. However, SMSr‐SAMD moderately suppresses the activity of DGKζ in contrast to DGKδ.

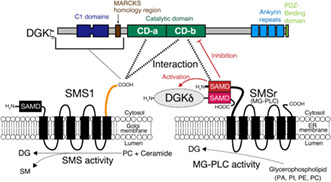

**Abbreviations**
CPES: ceramide phosphoethanolamine synthase
DG: diacylglycerol
DGK: diacylglycerol kinase
FL: full length
HRP: horseradish peroxidase
PA: phosphatidic acid
PAP: phosphatidic acid phosphatase
PC: phosphatidylcholine
PE: phosphatidylethanolamine
PI: phosphatidylinositol
PLC: phospholipase C
PS: phosphatidylserine
SAMD: sterile α motif domain
SMS: sphingomyelin synthase
SMSr: sphingomyelin synthase‐related protein
TEV: tobacco etch virus
TMD: transmembrane domain
TS: Twin‐Strep

Diacylglycerol kinase (DGK) phosphorylates diacylglycerol (DG) to convert it to phosphatidic acid (PA) [1, 2, 3, 4, 5]. Mammalian DGK consists of 10 isoforms, which can be divided into five groups: type I (α, β and γ), type II (δ, η and κ), type III (ε), type IV (ζ and ι), and type V (θ) [1, 2, 3, 4, 5]. In addition, there are several splice variants, such as DGKδ1 and 2 [6] and DGKη1 and 2 [7]. DG binds to and regulates C1 domain‐equipped proteins, such as conventional protein kinase C (PKC) and novel PKC and Ras guanine nucleotide releasing protein (GRP) [8, 9, 10, 11]. Moreover, various PA‐binding proteins (more than 70) have been reported, including C‐Raf, cAMP phosphodiesterase 4A1, atypical PKC (PKCζ), novel PKC (PKCδ and ε), sporulation‐specific protein 20p, Opi1p, α‐synuclein, Praja‐1, synaptojanin‐1, l‐lactate dehydrogenase A, and creatine kinase muscle type [12, 13, 14, 15, 16].

Diacylglycerol kinase isozymes function in a wide variety of physiological events, including immunity, cell proliferation, and the central nervous system [17, 18]. For example, DGKδ (type II) [19] regulates the epidermal growth factor receptor pathway in epithelial cells [20] and insulin receptor signaling in skeletal muscle [21, 22, 23]. Moreover, brain‐specific knockout (KO) of DGKδ, which enhances the serotonin nervous system through attenuation of serotonin transporter in the brain [24, 25, 26], leads to obsessive–compulsive disorder‐like behaviors [27]. DGKζ (type IV) [28, 29] reduces nuclear DG levels by shuttling between the nucleus and the cytoplasm and attenuates cell proliferation [30]. DGKζ functions as an immunosuppressor in T cells [31, 32]. DGKζ acts downstream of the leptin signaling pathway in the hypothalamus [33]. DGKζ promotes neurite outgrowth in NIE‐115 neuroblastoma cells [34]. DGKζ induced neurite outgrowth in a retinoic acid‐dependent and serum starvation‐dependent manner in Neuro‐2a neuroblastoma cells [35].

There are three isoforms of sphingomyelin synthase (SMS) [36, 37], SMS1 [38, 39], SMS2 [38], and SMS‐related protein (SMSr) [38]. SMS1, SMS2, and SMSr have six transmembrane domains (TMDs) and four conserved motifs, two of which are similar to the phosphatase domains in lipid phosphate phosphatase, and localize to the Golgi apparatus, the Golgi apparatus/plasma membrane, and the endoplasmic reticulum, respectively [36, 37]. The SMS1 and SMS2 proteins produce DG and sphingomyelin through the transfer of phosphocholine from PC to ceramide [36, 37]. SMSr has no SMS activity but exhibits ceramide phosphoethanolamine synthase (CPES) activity via the transfer of phosphoethanolamine from phosphatidylethanolamine (PE) to ceramide [40]. SMSs have various important roles in biological functions, such as cell proliferation, migration, apoptosis, and autophagy, and play roles in several human diseases, including cancer, cardiovascular disorders, and psychiatric disorders [36, 37, 41].

We recently searched for an upstream enzyme (a DG supply enzyme) of DGKδ. Consequently, we found that DGKδ interacts with SMSr [42], which showed PA phosphatase (PAP) and phosphatidylinositol (PI)‐/phosphatidylcholine (PC)‐phospholipase C (PLC) activities, instead of CPES activity, to generate DG [43] and that SMSr supplies DG to DGKδ [42]. Therefore, we hypothesized that other DGK isozymes (α, β, γ, η, κ, ε, ζ, ι, θ) also interact with the SMS isozymes, SMSr, SMS1, and SMS2.

In the present study, we comprehensively searched for interactions between DGK isozymes and SMS isozymes. We found that DGKζ binds to SMS1 and SMSr but not SMS2. Moreover, DGKζ interacts with SMSr and SMS1 in different manners. These results suggest that, beyond our expectations, DGK isozymes and SMS isozymes form a complex network.

## Materials and methods

### Materials

Mouse monoclonal anti‐V5 antibody (clone E10/V4RR, MA5‐15253) was obtained from Thermo Fisher Scientific (Waltham, MA, USA). Rabbit polyclonal anti‐GFP antibody (#598) was purchased from Medical and Biological Laboratories (Tokyo, Japan). Mouse monoclonal anti‐FLAG–tag antibody (F1804) was obtained from Sigma–Aldrich (St. Louis, MO, USA). Mouse monoclonal anti‐GFP antibody (sc‐9996) was purchased from Santa Cruz Biotechnology (Santa Cruz, CA, USA). Mouse monoclonal anti‐Strep II (M211‐3) and rabbit polyclonal anti‐GST (PM013) were obtained from Medical and Biological Laboratories (Nagoya, Japan). A horseradish peroxidase (HRP)‐conjugated goat anti‐mouse IgG antibody was obtained from Bethyl Laboratories (Montgomery, TX, USA). Goat anti‐rabbit IgG‐HRP was purchased from Jackson ImmunoResearch (West Grove, PA, USA).

Plasmids for expressing N‐terminal 3×FLAG‐tagged human or rat DGK isozymes [44] and for expressing C‐terminal V5‐tagged human SMS isoforms [42] in mammalian cells were used.

### Plasmid constructs

We used the following nomenclature for epitope‐tagged proteins: TagX‐(protein) and (protein)‐TagY means that TagX and TagY are located at the N and C termini of the protein, respectively.

The N‐terminal (NT) or C‐terminal (CT) cytosolic regions of SMS1 and SMSr were subcloned into the pAcGFP‐C1 vector (Clontech‐Takara Bio, Kusatsu, Japan) via In‐Fusion cloning (Clontech‐Takara Bio) at the *Eco*RI/*Sal*I sites. The amplicons were generated using the following primers: SMS1‐NT (aa 1–134) using 5′‐CTCAAGCTTCGAATTATGAAGGAAGTGGTTTATTG‐3′ (forward) and 5′‐CCGCGGTACCGTCGATCACTTGCCCCACTCCATG‐3′ (reverse); SMS1‐CT (aa 348–413) using 5′‐CTCAAGCTTCGAATTCACACTATGGCCAATCAGC‐3′ (forward) and 5′‐CCGCGGTACCGTCGATTATGTGTCATTCACCAGCC‐3′ (reverse); SMSr‐NT (aa 1–151), 5′‐CTCAAGCTTCGAATTATGGCAGGTCCTAATC‐3′ (forward) and 5′‐CCGCGGTACCGTCGATCACTTCCAGTATTCTGGGTC‐3′ (reverse); SMSr‐CT (aa 364–415), 5′‐CTCAAGCTTCGAATTCATACTCTGGCCAATACC‐3′ (forward) and 5′‐ CCGCGGTACCGTCGATCATCCAATTAGTCTTTTC‐3′ (reverse).

Glutathione *S*‐transferase (GST)‐tagged SMS1‐CT and SMSr‐NT were generated by in‐fusion cloning. The pGEX‐6P‐1 vector (GE Healthcare, Little Chalfont, UK) was linearized at the *Eco*RI and *Sal*I sites, and amplified gene with 15 bp extensions homologous to vector ends. We generated SMS1‐CT using 5′‐GGGATCCCCGGAATTCCACACTATGGCCAATCAGC‐3′ (forward) and 5′‐GTCGACCCGGGAATTCTATGTGTCATTCACCAGCC‐3′ (reverse) and SMSr‐NT using 5′‐GAATTCCCGGGTCGAATGGCAGGTCCTAATCAAC‐3′ (forward) and 5′‐GGCCGCTCGAGTCGATCACTTCCAGTATTCTGGGTCC‐3′ (reverse).

The cDNAs encoding DGK isozymes (α, β, γ, δ1, δ2, η1, η2, κ, ε, ζ, ι, and θ) that were subcloned into the expression plasmid, p3×FLAG‐CMV (Sigma–Aldrich), for expression in mammalian cells were generated as described [44].

N‐terminal 3×FLAG‐tagged human DGKζ mutants were generated by PCR and inserted into the *Eco*RI/*Sal*I sites of the 3×FLAG CMV 7.1 vector. The following DGKζ mutants were generated using the following primers: DGKζ‐NT (aa 1–283), 5′‐GGTGGTGAATTCAATGGAGCCGCGGGACGG‐3′ (forward) and 5′‐ACGCGTCGACCTAGAAGGGTCTCCAGCGGCC‐3′ (reverse); DGKζ‐CD (aa 284–640), 5′‐CCGGAATTCAATCATCAGGCCCACCCCC‐3′ (forward) and 5′‐ACGCGTCGACCTACACCGGCTGCTGGTCG‐3′ (reverse); DGKζ‐CD‐a (aa 284–641), 5′‐CCGGAATTCAATCATCAGGCCCACCCCC‐3′ (forward) and 5′‐ACGCGTCGACCTACTCAGGCCCTGCCTCGG‐3′ (reverse); DGKζ‐CD‐b (aa 433–640), 5′‐CCGGAATTCAGACCGAGATGAAGGCGCC‐3′ (forward) and 5′‐ACGCGTCGACCTACACCGGCTGCTGGTCG‐3′ (reverse); DGKζ‐CT (aa 641–928) using the primers 5′‐CCGGAATTCACCAGAGCAGTTGCGCATCC‐3′ (forward) and 5′‐ACGCGTCGACCTACACAGCCGTCTCCTGGTC‐3′ (reverse).

The plasmid expressing N‐terminal Tobacco Etch Virus (TEV) protease cleavable Twin‐Strep‐tag (ENLYFQGS‐WSHPQFEK‐(GGGS)_2_‐GGSA‐WSHPQFEK) was cloned into the *Xho*I/*Bgl*II site of the pCAGGS vector [45] to generate an N‐terminal TEV protease cleavable Twin‐Strep‐tagged protein expression vector. We designated the vector “pCAGGS‐C‐TEV‐Twin‐Strep”. Full‐length (FL) DGKζ was subcloned into the *Bgl*II site of pCAGGS‐C‐TEV‐Twin‐Strep via In‐Fusion cloning. The following primers were used to amplify FL DGKζ: forward, 5′‐TTTTCAAGGCAGATCTATGGAGCCGCGGGACG‐3′; reverse, 5′‐AGAGGGAAAAAGATCTCTACACAGCCGTCTCCTGG‐3′. N‐terminal Twin‐Strep‐tagged DGKζ was subcloned into pOET3 vector (Oxford Expression Technologies, Oxford, UK). The following primers were used, forward, 5′‐TTTTCAAGGCAGATCTATGGAGCCGCGGGACG‐3′; reverse, 5′‐TTATTAATTAAGATCTCTACACAGCCGTCTCCTGG‐3′.

### Cell culture and transfection

HEK293 cells (Japanese Collection of Research Bioresources, Tokyo, Japan) were maintained in Dulbecco's modified Eagle's medium (D‐MEM; Wako Pure Chemicals, Osaka, Japan) supplemented with 5% FBS (Thermo Fisher Scientific) and 100 U·mL^−1^ penicillin/100 μg·mL^−1^ streptomycin (Wako Pure Chemicals) at 37 °C in an atmosphere containing 5% CO_2_. The plasmids were transiently transfected using PolyFect (Qiagen, Hilden, Germany) according to the manufacturer's instructions or using polyethylenimine Max (#24765‐100; Polysciences, Warrington, PA, USA) [46]. The expression vectors with polyethylenimine Max (1 mg·mL^−1^, pH 8.0) were preincubated for 10 min at a 1 : 3 ratio (20 μg DNA: 60 μL polyethylenimine Max) in 750 μL of Opti‐MEM before transfection. The cells overexpressing recombinant proteins were harvested after 24 h and the pellets were resuspended in 40% (v/v) glycerol diluted in phosphate‐buffered saline. The cell samples were flash‐frozen in liquid nitrogen and stored at −80 °C until use.

Sf9 cells were maintained in Sf‐900 II serum‐free medium (Invitrogen, Waltham, MA, USA) in sterile Erlenmeyer flask at 120 r.p.m. and 28 °C without CO_2_ in the dark. Volume of the medium was kept at 20–30% of flask volume. To generate recombinant baculovirus was generated using pOET3 vector and the flashBAC system (Oxford Expression Technologies) as described previously [43].

### Immunoprecipitation

HEK293 cell lysates expressing V5‐tagged SMS1, SMS2, SMSr, or their AcGFP‐tagged mutants and 3×FLAG‐tagged DGKs (α, β, γ, δ, η, κ, ε, ζ, ι, θ) or their mutants were subjected to immunoprecipitation with anti‐V5 (MA5‐15253) or anti‐GFP (#598) antibody and Protein A/G PLUS‐agarose beads (Santa Cruz Biotechnology) as described previously [42].

### GST pull‐down assay

Glutathione *S*‐transferase‐fused SMS1‐CT and SMSr‐NT were bacterially expressed and highly purified using glutathione‐Sepharose beads (GE Healthcare). Twin‐Strep (TS)‐tagged DGKζ (TS‐DGKζ) was expressed by mammalian cells and highly purified using Strep‐Tactin XT beads (IBA Lifesciences, Goettingen, Germany).

Glutathione *S*‐transferase pull‐down assays were performed as previously [42]. Purified GST‐SMS1‐CT or SMSr‐NT were incubated with glutathione‐Sepharose beads for 30 min at 4 °C with constant rocking. The beads were washed five times with buffer containing 20 mm Tris–HCl, pH 7.5, 150 mm NaCl, 1 mm EDTA, 0.1% (v/v) Triton X‐100, and 1 mm phenylmethylsulfonyl fluoride. Purified Twin‐strep tagged DGKζ was incubated with the beads for 2 h at 4 °C with constant rocking. Then, the beads were washed five times with buffer. The washed beads washed were then boiled in SDS sample buffer, and the extracts were analyzed by western blotting.

### Mammalian cell expression and purification of Twin‐Strep (TS)‐tagged proteins

C‐terminally TS‐tagged human SMS1 and SMSr (SMS1‐TS and SMSr‐TS), and N‐terminally TS‐tagged DGKζ (TS‐DGKζ) were expressed in HEK293 cells. SMS1‐TS and SMSr‐TS were lysed via lysed via homogenization on ice with ice‐cold lysis buffer (20 mm Tris–HCl, pH 7.4, containing 150 mm NaCl, 10% (v/v) glycerol, 1 mm PMSF, 0.1 mm DTT, 20 μg·mL^−1^ aprotinin, 20 μg·mL^−1^ leupeptin, 20 μg·mL^−1^ pepstatin, and 20 μg·mL^−1^ soybean trypsin inhibitor) containing detergents (1% (w/v) *n*‐dodecyl‐β‐d‐maltoside (DDM) and 0.2% (w/v) cholesteryl hemisuccinate (CHS)). The supernatant (1% DDM soluble fraction) was isolated by ultracentrifugation at 100 000 ***g*** for 30 min at 4 °C and then purified using Strep‐Tactin XT beads. The beads were washed with the lysis buffer containing 0.05% DDM and 0.01% CHS. Subsequently, the bound proteins were eluted with the lysis buffer containing 0.05% DDM, 0.01% CHS, and 2.5 mm d‐desthiobiotin (Sigma–Aldrich). TS‐tagged DGKζ was purified using Strep‐Tactin XT beads without detergents.

### DGK activity assay

Diacylglycerol kinase activity was measured using liquid chromatography–tandem mass spectrometry (LC–MS/MS) as previously described [42].

### Western blotting

Western blotting was carried out as previously described [42]. Equal quantities of protein were loaded onto a polyacrylamide gel. Separated proteins were transferred onto a polyvinylidene fluoride membrane (Millipore, Burlington, MA, USA) and incubated overnight at 4 °C with the following primary antibodies: anti‐FLAG (F‐1804), V5 (MA5‐15253), GFP (sc‐9996), Twin‐Strep (M211‐3), and GST (PM013) antibodies. After washing, the membranes were incubated with a secondary antibody solution (goat anti‐rabbit IgG‐HRP or goat anti‐mouse IgG‐HRP) at room temperature for 1 h, followed by detection using the enhanced chemiluminescence method.

### Statistical analysis

Data are represented as the means ± SDs and were analyzed by the Student's *t* test for the comparison of two groups or one‐way ANOVA followed by Tukey's or Dunnett's *post hoc* test for multiple comparisons using graphpad prism 8 (GraphPad Software, Boston, MA, USA) to determine any significant differences. *P* < 0.05 was considered significant.

## Results

### DGKζ interacts with SMSr and SMS1

**Fig. 1:**
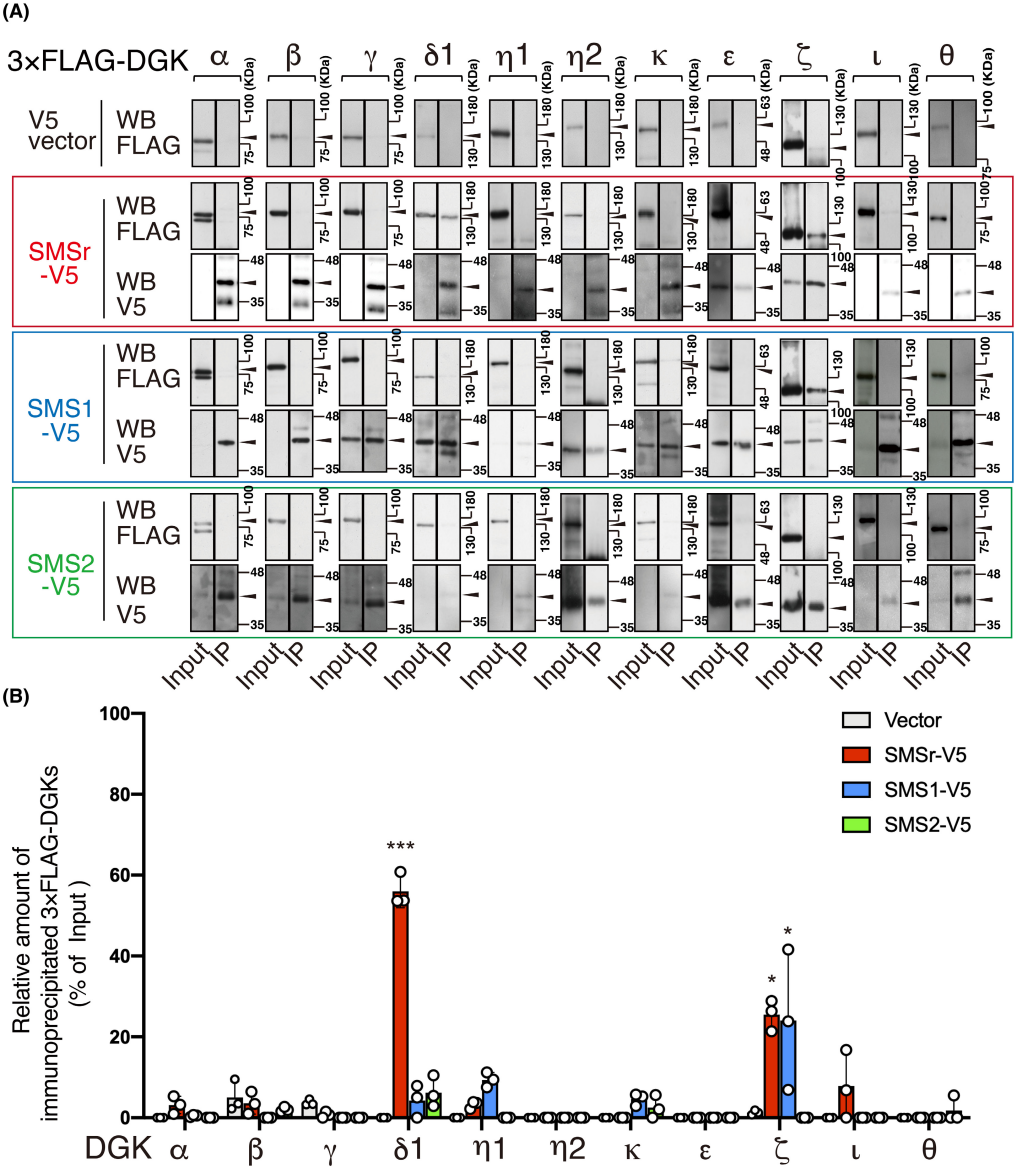
Identification of DGK isozymes interacting with SMSr, SMS1, and SMS2. (A) Immunoprecipitation (IP)‐western blot (WB) analysis of the interaction of vector alone (V5 vector), SMSr‐V5, SMS1‐V5, and SMS2‐V5 with 3×FLAG‐DGK isozymes (α, β, γ, δ1, δ2, η1, η2, κ, ε, ζ, ι, and θ). V5‐tagged protein was immunoprecipitated with an anti‐V5 antibody. SDS/PAGE (10% acrylamide) was performed, and separated proteins were detected by western blotting with anti‐FLAG and anti‐V5 antibodies. A representative of three repeated experiments is shown. Left panel, cell lysate (Input); right panel, IP. (B) Densitometric quantification of 3×FLAG‐tagged DGKs using imagej fiji software (US National Institutes of Health, Bethesda, MD, USA). Binding activity was calculated as the percentage of the band intensity in the IP sample compared with the input band intensity. The values are presented as the means ± SD of three independent experiments. *P < 0.05, ***P < 0.005 versus vector alone.

We first examined the interaction of all DGK isozymes with SMSr (Fig. 1). We confirmed that 3×FLAG‐tagged DGK isozymes in V5 vector alone‐expressing cells (without SMSr‐V5, mock) failed to be precipitated by an anti‐V5 antibody (Fig. 1A,B). As previously demonstrated for DGKδ2 [42], DGKδ1 was coimmunoprecipitated with SMSr (Fig. 1A,B). Notably, we found that, in addition to DGKδ1 and δ2, DGKζ was also strongly cosedimented with SMSr (Fig. 1A,B). Other DGK isozymes (α, β, γ, η1, η2, κ, ε, ι, and θ) failed to show such cosedimentation (Fig. 1A,B).

Next, we determined the interaction between 10 DGK isozymes and SMS1 (Fig. 1A,B). As previously reported for DGKδ2 [42], DGKδ1 did not coimmunoprecipitate with SMS1 (Fig. 1A,B). Intriguingly, we found that DGKζ strongly coimmunoprecipitated with SMS1 (Fig. 1A,B) in addition to SMSr. However, other DGK isozymes (α, β, γ, η1, η2, κ, ε, ι, and θ) failed to exhibit such coimmunoprecipitation (Fig. 1A,B).

We next examined the interaction between 10 DGK isozymes and SMS2 (Fig. 1A,B). As previously reported for DGKδ2 [42], DGKδ1 was not coimmunoprecipitated with SMS2 (Fig. 1A,B). Moreover, no marked coimmunoprecipitation of other DGK isozymes (α, β, γ, η1, η2, κ, ε, ζ, ι, θ), including DGKζ, was observed (Fig. 1A,B). Taken together, these results indicate that DGKζ selectively interacts with SMSr and SMS1, although DGKδ interacts with only SMSr [42].

### The N‐terminal SAMD of SMSr and the C‐terminal region of SMS1 interact with DGKζ

**Fig. 2:**
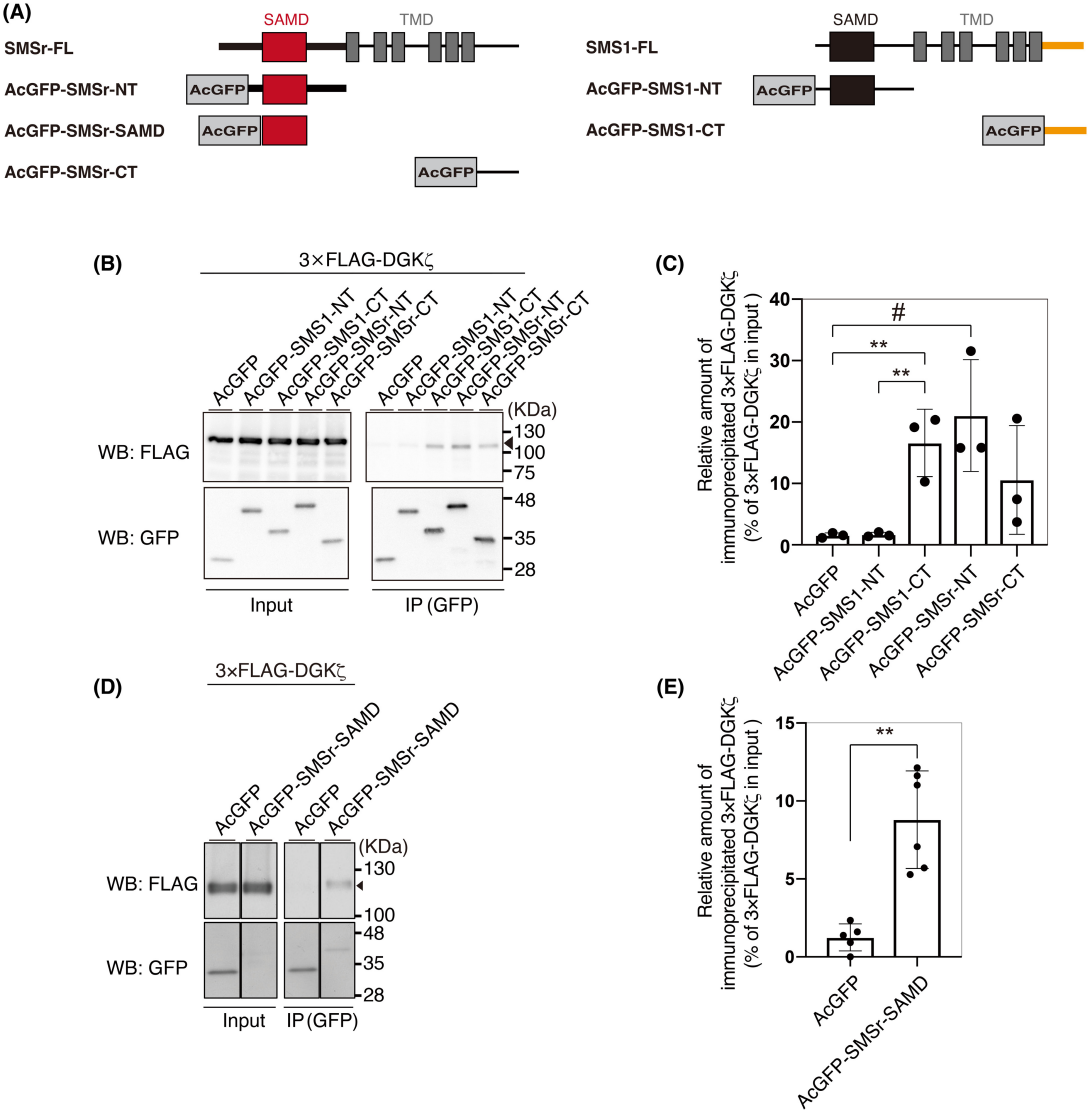
DGKζ‐binding activities of SMSr‐NT, SMS1r‐CT, SMSr‐SAMD, SMS1‐NT, and SMS1‐CT. (A) Schematic representation of the structures of AcGFP‐tagged SMSr‐NT, SMS1r‐CT, SMSr‐SAMD, SMS1‐NT, and SMS1‐CT. (B, D) Immunoprecipitation (IP)‐western blot (WB) analysis of the interaction of 3×FLAG‐tagged DGKζ‐FL with AcGFP‐tagged SMSr‐NT, SMSr‐CT, SMS1‐NT and SMS1‐CT (B) and SMSr‐SAMD (D). AcGFP‐tagged proteins were immunoprecipitated with an anti‐GFP antibody. SDS/PAGE (10% acrylamide) was performed, and separated proteins were detected by Western blotting with anti‐GFP and anti‐FLAG antibodies. (C, E) Quantitative analysis of western blotting by densitometry was performed using imagej fiji software. The values are presented as the means ± SD of three independent experiments. (C) **P<0.01, among AcGFP, AcGFP‐SMS1‐NT and AcGFP‐SMS1‐CT, #P<0.05, among AcGFP, AcGFP‐SMSr‐NT and AcGFP‐SMSr‐CT; (E)   **P < 0.01.

We next attempted to determine a DGKζ‐interaction region in SMSr. AcGFP‐tagged N‐terminal (AcGFP‐SMSr‐NT) and C‐terminal (AcGFP‐SMSr‐CT) cytosolic regions of SMSr were generated (Fig. 2A), and their association with 3×FLAG‐tagged DGKζ was determined. We found that the N‐terminal cytosolic region of SMSr, which contains SAMD, strongly interacted with DGKζ (Fig. 2B,C). Although the C‐terminal cytosolic region of SMSr moderately cosedimented DGKζ, statistical significance was not detected (Fig. 2B,C).

A DGKζ‐interaction region in SMS1 was also identified. For this purpose, AcGFP‐tagged N‐terminal (AcGFP‐SMS1‐NT) and C‐terminal (AcGFP‐SMS1‐CT) cytosolic regions of SMS1 were made (Fig. 2A). Interestingly, unlike SMSr, the C‐terminal cytosolic region of SMS1, but not the N‐terminal cytosolic region, was strongly associated with DGKζ (Fig. 2B,C).

To narrow the DGKζ‐interaction area in the N‐terminal cytosolic region of SMSr, the DGKζ‐interaction activity of SAMD alone of SMSr was tested. Figure 2D,E, 3D,E show that the SAMD of SMSr bound to DGKζ.

**Fig. 3:**
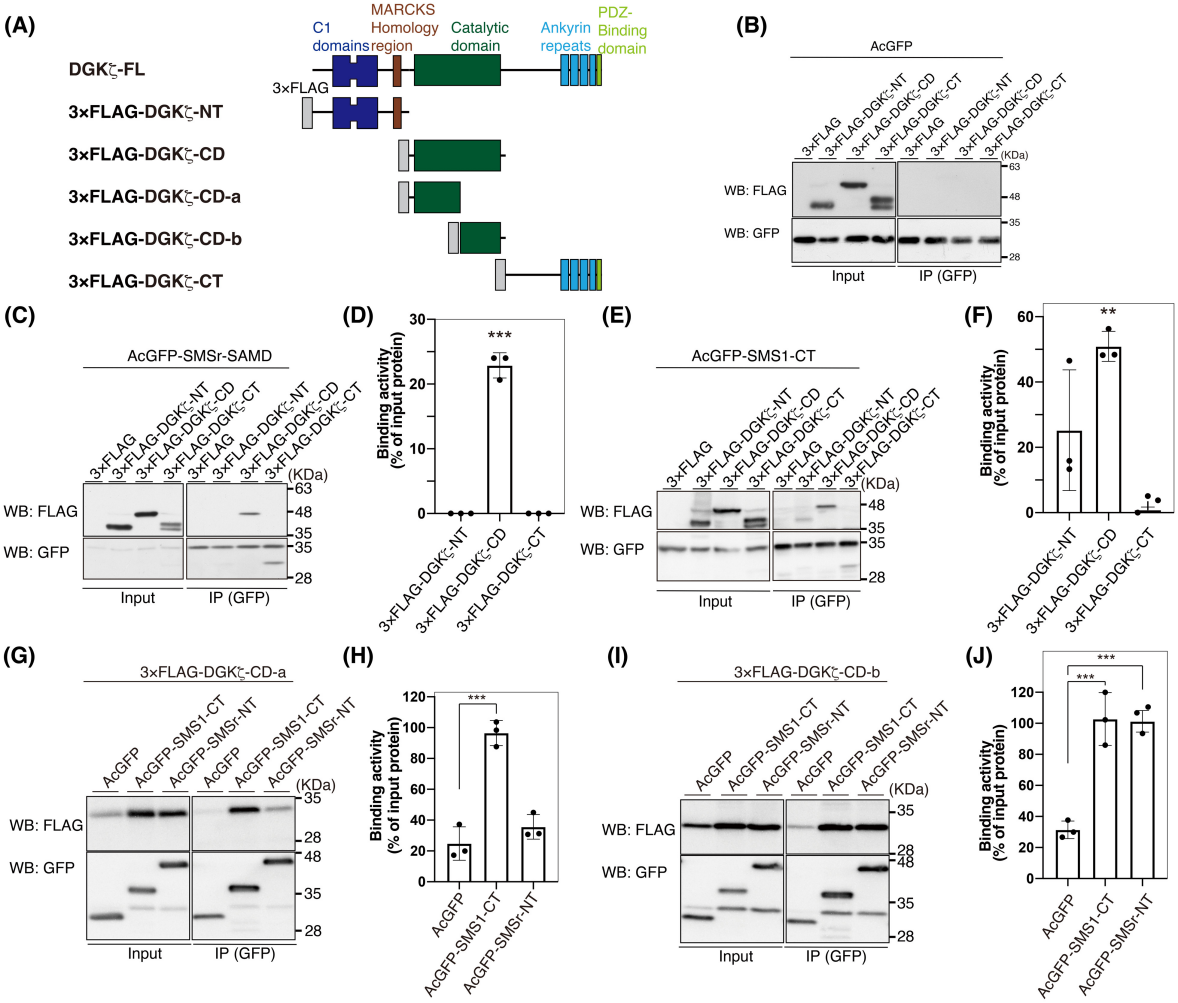
Binding activities of various DGKζ deletion mutants to SMSr‐SAMD and SMS1‐CT. (A) Schematic representation of the structures of 3×FLAG‐tagged DGKζ deletion mutants. (B, C, E, G, J) Immunoprecipitation (IP)‐western blot (WB) analysis of the interaction of AcGFP alone with 3×FLAG‐tagged DGKζ‐NT, DGKζ‐CD, and DGKζ‐CT (B); the interaction of AcGFP‐tagged SMSr‐SAMD with 3×FLAG‐tagged DGKζ‐NT, DGKζ‐CD and DGKζ‐CT (C); the interaction of AcGFP‐tagged SMS1‐CT with 3×FLAG‐tagged DGKζ‐NT, DGKζ‐CD and DGKζ‐CT (E); the interaction of AcGFP‐tagged SMSr‐NT and SMS1‐CT with 3×FLAG‐tagged DGKζ‐CD‐a (G); and the interaction of AcGFP‐tagged SMSr‐NT and SMS1‐CT with 3×FLAG‐tagged DGKζ‐CD‐b (I). AcGFP‐tagged SMSr‐SAMD, SMSr‐NT or SMS1‐CT was immunoprecipitated with an anti‐GFP antibody. SDS/PAGE (12% acrylamide) was performed, and separated proteins were detected by western blotting with anti‐GFP and anti‐FLAG antibodies. (D, F, H, J) Quantitative analysis of western blotting by densitometry was performed using imagej fiji software [51]. Binding activity was calculated as the percentage of the band intensity in the IP sample compared with the input band intensity. The values are presented as the means ± SD of three independent experiments. **P < 0.01, ***P < 0.005 versus AcGFP alone.

### The catalytic domain of DGKζ interacts with SMSr and SMS1

To determine an SMSr‐interaction region in DGKζ, we divided the protein into three parts, DGKζ‐NT, DGKζ‐CD, and DGKζ‐CT (Fig. 3A), and determined their interaction with SMSr‐SAMD. We confirmed that they failed to bind to AcGFP alone (Fig. 3B). As shown in Fig. 3C,D, 3×FLAG‐DGKζ‐CD, but not 3×FLAG‐DGKζ‐NT or 3×FLAG‐DGKζ‐CT, strongly interacted with AcGFP‐SMSr‐SAMD.

Next, the interaction of DGKζ‐NT, DGKζ‐CD, and DGKζ‐CT with SMS1‐CT was examined. Among them, 3×FLAG‐DGKζ‐CD most strongly interacted with AcGFP‐SMS1‐CT (Fig. 3E,F). Although 3×FLAG‐DGKζ‐NT was also cosedimented with AcGFP‐SMS1‐CT (Fig. 3E,F), statistical significance was not detected (Fig. 3F).

We further divided the catalytic domain of DGKζ into CD‐a and CD‐b (Fig. 3A) and determined their interaction with SMSr‐NT and SMS1‐CT. 3×FLAG‐DGKζ‐CD‐a strongly interacted with only AcGFP‐SMS1‐CT (Fig. 3G,H). Unlike 3×FLAG‐DGKζ‐CD‐a, 3×FLAG‐DGKζ‐CD‐b bound to both AcGFP‐SMSr‐NT and AcGFP‐SMS1‐CT, indicating that SMSr‐NT interacts with DGKζ‐CD‐a and that SMS1‐CT binds to both DGKζ‐CD‐a and DGKζ‐CD‐b (Fig. 3I, J).

### DGKζ directly interacts with SMSr and SMS1

**Fig. 4:**
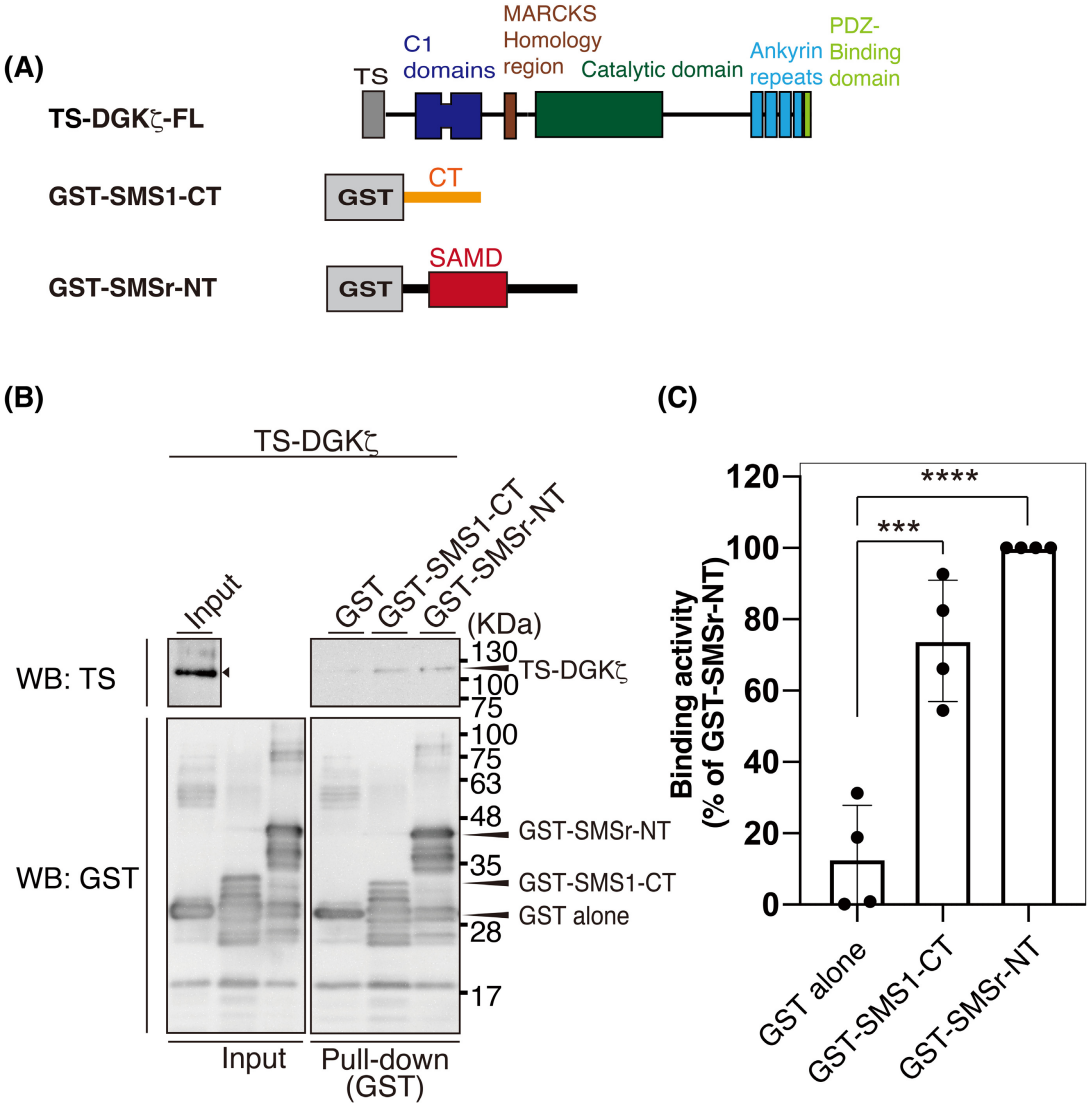
Binding activities of purified DGKζ with SMS1‐CT and SMSr‐NT. (A) Schematic representation of the structures of Twin‐Strep (TS)‐tagged DGKζ (TS‐DGKζ), GST‐tagged SMS1‐CT, and GST‐tagged SMSr‐NT. (B) The interaction of TS‐DGKζ with GST‐SMS1‐CT and GST‐SMSr‐NT. GST‐SMS1‐CT or GST‐SMSr‐NT was pulled down with glutathione‐Sepharose beads. SDS/PAGE (12% acrylamide) was performed and separated proteins were detected by western blotting with anti‐GST and anti‐TS antibodies. (C) Quantitative analysis of western blotting by densitometry was performed using imagej fiji software. Binding activity was calculated as the percentage of the band intensity (TS‐DGKζ) in the pull‐down sample compared with that of GST‐SMSr‐NT‐pull down (set to 100). The values are presented as the means ± SD of three independent experiments. ***P < 0.005, ****P < 0.001 versus GST alone.

We next examined whether DGKζ directly binds to SMSr and SMS1. GST‐fused SMSr‐NT and SMS1‐CT (Fig. 4A) were bacterially expressed and purified. Smaller bands of GST‐SMSr‐NT and GST‐SMS1‐CT, likely degradation products of GST‐SMSr‐NT and GST‐SMS1‐CT, were detected (Fig. 4B). Twin‐Strep (TS)‐tagged DGKζ (TS‐DGKζ) was also expressed in HEK293 cells and purified. As shown in Fig. 4B,C, purified GST‐SMSr‐NT and GST‐SMS1‐CT strongly pulled down purified TS‐DGKζ. These results indicate that DGKζ directly interacts with SMSr and SMS1.

### SMSr inhibits DGKζ activity

**Fig. 5:**
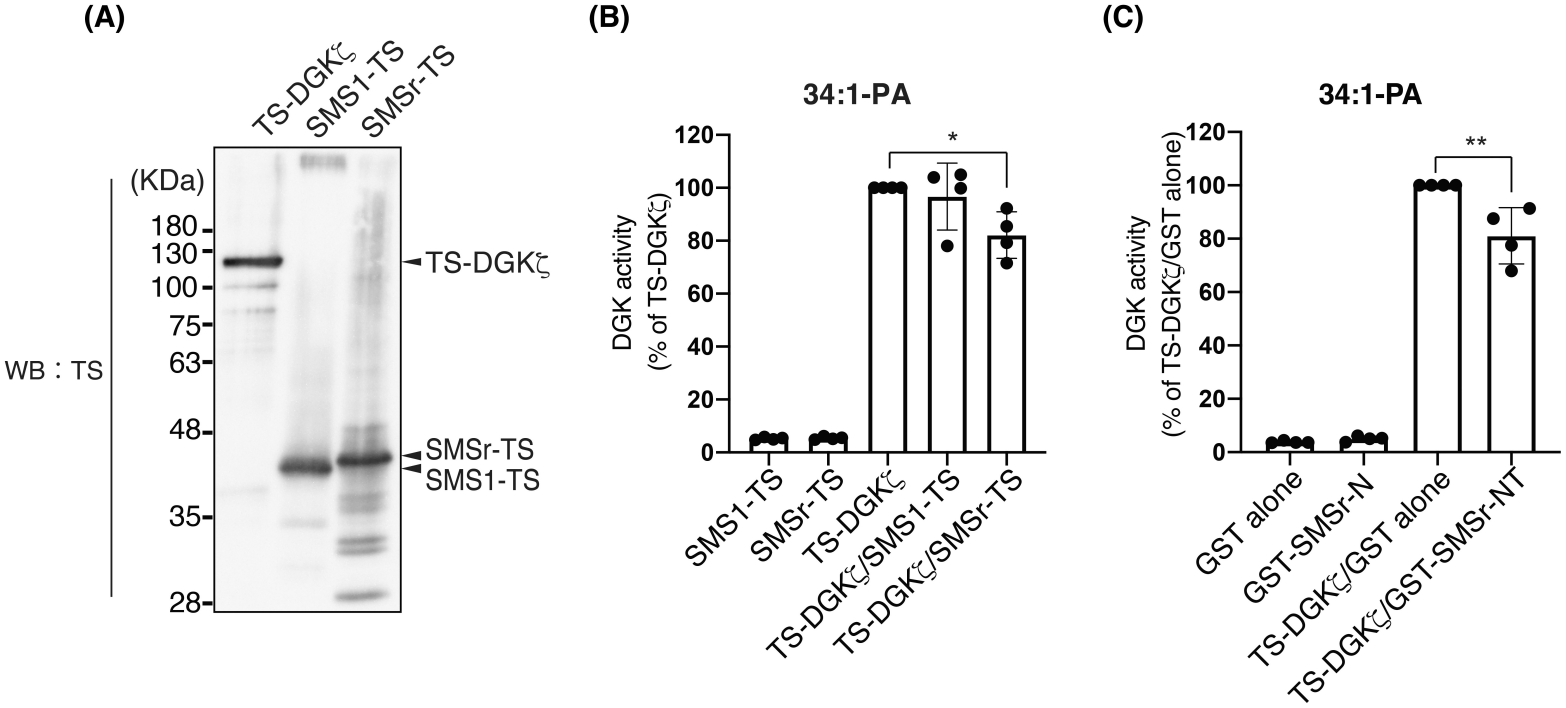
Effects of SMSr and SMS1 on DGKζ activity *in vitro*. (A) Purification of TS‐DGKζ, SMS1‐TS, and SMSr‐TS. TS‐DGKζ, SMS1‐TS, and SMSr‐TS were expressed in Sf9 insect cells and purified using Strep‐Tactin XT beads. Purified TS‐DGKζ, SMS1‐TS, and SMSr‐TS were detected by immunoblot with anti‐TS antibody. (B) Effects of SMS1 and SMSr on DGKζ activity *in vitro* in the presence of 34:1 (16:0/18:1)‐DG. The activities of DGKζ (34:1 (16:0/18:1)‐PA production) were measured using LC–MS/MS. The activity of TS‐DGKζ alone was set to 100. The values are presented as the means ± SD of four independent experiments. *P < 0.05 versus TS‐DGKζ alone. (C) Effects of SMSr‐NT on DGKζ activity *in vitro* in the presence of 34:1 (16:0/18:1)‐DG. The activities of DGKζ (34:1 (16:0/18:1)‐PA production) were measured using LC–MS/MS. The activity of TS‐DGKζ alone was set to 100. The values are presented as the means ± SD of four independent experiments. **P < 0.01 versus TS‐DGKζ/GST alone.

We previously reported that SMSr interacted with DGKδ2 via their SAMDs and activated DGKδ2 (more than 2‐fold) *in vitro* [42]. Moreover, SMSr‐NT and SMS1‐CT interacted with CD (catalytic domain)‐b and CD‐a/b, respectively (Fig. 4). Therefore, we analyzed the effects of purified SMSr‐TS and SMS1‐TS (Fig. 5A) on the activity of purified TS‐DGKζ (Fig. 5A) *in vitro* in the presence of 34:1 (16:0/18:1)‐DG. Intriguingly, SMSr, but not SMS1, moderately inhibited DGKζ activity (Fig. 5B). Moreover, purified GST‐SMSr‐NT including SAMD (see Fig. 4B) also attenuated the activity of purified DGKζ (Fig. 5C). These results indicate that SMSr moderately suppresses the activity of DGKζ in contrast to DGKδ2 [42].

## Discussion

In the present study, we demonstrated for the first time that DGKζ interacts with SMS1 and SMSr but not SMS2 (Figs 1 and 5). DGKδ1 and δ2 also bound to only SMSr but not SMS1 or SMS2 (Figs 1 and 5), as previously reported [42]. Moreover, DGKα, β, γ, η1, η2, κ, ε, ι, and θ failed to show interactions with SMSr and SMS1 (Fig. 1). Therefore, the interaction between DGKζ and SMSr and the association between DGKζ and SMS1 are highly selective.

We previously reported that DGKδ associates with SMSr via the interaction between DGKδ‐SAMD and SMSr‐SAMD [42]. Although DGKζ does not have SAMD [1, 2, 3, 4, 5], unlike DGKδ, the protein interacted with SMSr. Notably, the interaction occurred between DGKζ‐CD‐b and SMSr‐SAMD (Figs 2 and 3). We searched for a SAMD‐like region in CD‐b of DGKζ. However, such a region was not found. Because SAMD has two interfaces to form oligomer structures [47], DGKζ‐CD‐b may interact with another interface of SMSr‐SAMD, which is different from the SMSr‐SAMD–DGKδ‐SAMD interface. However, the binding mechanisms between DGKζ‐CD‐b and SMSr‐SAMD are still unclear.

**Fig. 6:**
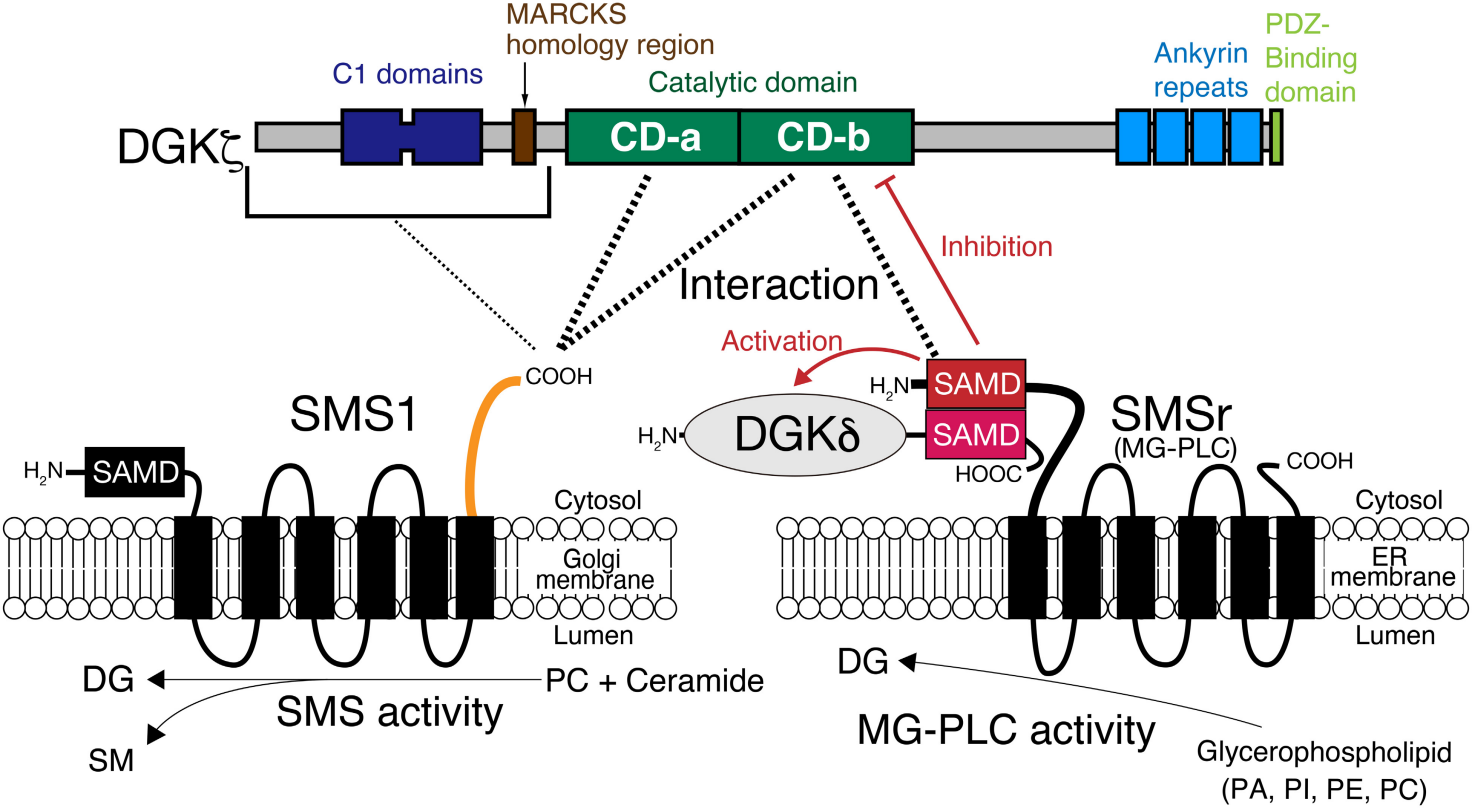
Schematic representation of the interaction of DGKζ with SMS1 and SMSr. SMSr‐SAMD binds to DGKζ‐CD‐b. SMS1‐CT interacts with DGKζ‐CD‐a and DGKζ‐CD‐b. We previously demonstrated that DGKδ‐SAMD associates with SMSr‐SAMD and that SMSr activates DGKδ [42]. See Results and Discussion for the full description. MG‐PLC, multiglycerophospholipid PLC hydrolase [43].

SMSr, but not SMS1, inhibited DGKζ activity (Fig. 5). Therefore, it is likely that DGKζ efficiently phosphorylates DG supplied from SMS1, while DGKζ may not effectively utilize DG provided by SMSr. We previously demonstrated that SMSr enhanced DGKδ activity (more than 2‐fold) [42] (Fig. 6). Therefore, it is possible that, as a biological function, SMSr regulates the balance of DGKδ‐ and DGKζ‐activities in addition to DG supply to these DGK isozymes.

In the case of the SMSr–DGKζ interaction, SAMD in the N‐terminal cytosolic region of SMSr binds to DGKζ (Fig. 2). In contrast, SMS1 uses its C‐terminal cytosolic region, which does not contain SAMD, for the SMS1–DGKζ interaction (Fig. 2). Therefore, SMSr and SMS1 utilize different regions for the same target, DGKζ. SMS1 also has SAMD at the N terminus. The amino acid sequences of SMSr‐SAMD and SMS1‐SAMD are considerably different from each other (identity: 30.9%; similarity: 48.5%) (Fig. S1); this moderate difference could confer the selectivity of SMSr‐SAMD–DGKζ binding. When comparing the amino acid sequences of the C‐terminal cytosolic regions of SMSr, SMS1 and SMS2, there are considerable differences among them (identity (SMS1 vs. SMSr): 36.4%; similarity (SMS1 vs. SMSr): 48.5%; identity (SMS1 vs. SMS2): 41.9%; similarity (SMS1 vs. SMS2): 58.1%) (Fig. S2). These differences could explain the selectivity of the SMS1‐CT–DGKζ association.

SMSr‐SAMD interacted with DGKζ‐CD‐a (Fig. 3). However, SMS1‐CT bound to DGKζ‐CD‐a and CD‐b (Fig. 3). Therefore, SMSr‐SAMD and SMS1‐CT associate with different regions of DGKζ, although they partly overlap. When the amino acid sequences of CD‐a and CD‐b in DGKζ were compared with those of CD‐a and CD‐b in DGKι, which is most closely related to DGKζ [1, 2, 3, 4, 5], there were modest differences (identity: 81.2%; similarity: 90.6%) (Fig. S3). These differences likely generate the selectivity of the SMSr‐SAMD–DGKζ and SMS1‐CT–DGKζ association. DGKζ is known to have alternative splicing products, DGKζ1 [28] and ζ2 [48], which contain different N‐terminal sequences. In the present study, DGKζ1 (104 kDa) [28] was used. Because SMSr and SMS1 bound to DGKζ‐CD‐a and CD‐b in a DGKζ N‐terminal sequence‐independent manner (Fig. 3), both DGKζ1 and ζ2 likely interact with SMSr and SMS1.

Although SMSr is a DG‐generating enzyme, its CPES activity is very low [43]. We recently found that SMSr has high PAP, PI‐PLC, PE‐PLC, and PC‐PLC activities, which produce DG, instead of CPES activity [43]. SMS1 generates DG and sphingomyelin through the transfer of phosphocholine from PC to ceramide [36, 37]. DG is known to quickly diffuse across the lipid bilayer by flip‐flop [49]. Therefore, it is considered that the DG generated by SMSr and SMS1 immediately transverses the Golgi and endoplasmic reticulum membranes from the lumen side to the cytosol leaflet and, consequently, is supplied to DGKζ, which exists in the cytoplasm, as illustrated in Fig. 6. Moreover, DGKζ (https://www.proteinatlas.org/ENSG00000149091‐DGKZ/tissue), SMSr (https://www.proteinatlas.org/ENSG00000156671‐SAMD8/tissue), and SMS1 (https://www.proteinatlas.org/ENSG00000198964‐SGMS1/tissue) are ubiquitously expressed in a variety of tissues [50]. These results indicate that DGKζ and SMSr/SMS1 can functionally link to each other.

In summary, in the present study, we demonstrated for the first time that DGKζ interacts with SMS1 and SMSr but not SMS2 (Fig. 6). DGKδ also associates with SMSr via their SAMDs [42] (Fig. 6). Therefore, it is likely that DGK isozymes and SMS isozymes form a complex network. Intriguingly, DGKζ interacts with SMS1 and SMSr in different manners. These data suggest that SMSr and SMS1 are promising candidates for DG supply enzymes upstream of DGKζ and that they compose novel and distinct DG‐signaling pathways. However, further studies will be required to analyze whether SMS1 and SMSr are functionally linked to DGKζ. Moreover, we need to find candidates for DG supply enzymes upstream of other isozymes (eight isozymes: α, β, γ, η, κ, ε, ι, and θ).

## Conflict of interest

The authors declare no conflict of interest.

### Peer review

The peer review history for this article is available at https://www.webofscience.com/api/gateway/wos/peer‐review/10.1002/2211‐5463.13628.

## Author contributions

MF primarily designed and conducted the experiments and analyzed the data. CM, YN, and RS designed and conducted the experiments and analyzed the data. CM, FS, and MF wrote the manuscript. CM and FS conceived the research. All authors revised the manuscript and approved its final version.

## Supporting information


**Fig. S1.** Sequence alignment of SMSr‐SAMD and SMS1‐SAMD. (A) Sequence alignment of SMSr‐SAMD (aa 12–78) and SMS1‐SAMD (aa 7–70). Sequence alignment was created using Clustal Omega provided by EMBL's European Bioinformatics Institute (EMBL‐EBI). Compared with SMSr‐SAMD, white letters on a black background indicate fully conserved residues, and black letters on a gray background indicate strongly similar residues. (B) Amino acid identities between the SAMDs of SMSr and SMS1. Amino acid identity and similarity were determined using Pairwise Sequence Alignment provided by the European Molecular Biology Open Software Suite (EMBOSS).
**Fig. S2.** Multiple sequence alignment of the C‐terminal regions of SMS1, SMS2, and SMSr. (A) Multiple sequence alignment of the C‐terminal regions of SMS1‐CT (aa 348–413), SMS2‐CT (aa 292–365), and SMSr‐CT (aa 364–415). Multiple sequence alignment was created using Clustal Omega provided by EMBL's European Bioinformatics Institute (EMBLEBI). Compared with SMS1‐CT, white letters on a black background indicate fully conserved residues, and black letters on a gray background indicate strongly similar residues. (B) Amino acid identities between the C‐terminal regions of SMS1, SMS2, and SMSr. Amino acid identity and similarity were determined using Pairwise Sequence Alignment provided by the European Molecular Biology Open Software Suite (EMBOSS).
**Fig. S3.** Sequence alignment of DGKζ‐CD and DGKι‐CD. (A) Sequence alignment of DGKζ‐CD (aa 293–622) and DGKι‐CD (aa 374–702). Sequence alignment was created using Clustal Omega provided by EMBL's European Bioinformatics Institute (EMBL‐EBI). Compared with DGKζ‐CD, white letters on a black background indicate fully conserved residues, and black letters on a gray background indicate strongly similar residues. (B) Amino acid identities between DGKζ‐CD and DGKι‐CD. Amino acid identity and similarity were determined using Pairwise Sequence Alignment provided by the European Molecular Biology Open Software Suite (EMBOSS).

## Data Availability

The data that support the findings of this study are available from the corresponding authors upon reasonable request.

## References

[1] Goto K , Nakano T and Hozumi Y (2006) Diacylglycerol kinase and animal models: the pathophysiological roles in the brain and heart. Adv Enzyme Regul 46, 192–202. 16854450 10.1016/j.advenzreg.2006.01.005

[2] Merida I , Avila‐Flores A and Merino E (2008) Diacylglycerol kinases: at the hub of cell signalling. Biochem J 409, 1–18. 18062770 10.1042/BJ20071040

[3] Sakane F , Imai S , Kai M , Yasuda S and Kanoh H (2007) Diacylglycerol kinases: why so many of them? Biochim Biophys Acta 1771, 793–806. 17512245 10.1016/j.bbalip.2007.04.006

[4] Sakane F , Mizuno S , Takahashi D and Sakai H (2018) Where do substrates of diacylglycerol kinases come from? Diacylglycerol kinases utilize diacylglycerol species supplied from phosphatidylinositol turnover‐independent pathways. Adv Biol Regul 67, 101–108. 28918129 10.1016/j.jbior.2017.09.003

[5] Topham MK and Epand RM (2009) Mammalian diacylglycerol kinases: molecular interactions and biological functions of selected isoforms. Biochim Biophys Acta 1790, 416–424. 19364481 10.1016/j.bbagen.2009.01.010 PMC2744455

[6] Sakane F , Imai S , Yamada K , Murakami T , Tsushima S and Kanoh H (2002) Alternative splicing of the human diacylglycerol kinase δ gene generates two isoforms differing in their expression patterns and in regulatory functions. J Biol Chem 277, 43519–43526. 12200442 10.1074/jbc.M206895200

[7] Murakami T , Sakane F , Imai S , Houkin K and Kanoh H (2003) Identification and characterization of two splice variants of human diacylglycerol kinase η. J Biol Chem 278, 34364–34372. 12810723 10.1074/jbc.M301542200

[8] Hurley JH , Newton AC , Parker PJ , Blumberg PM and Nishizuka Y (1997) Taxonomy and function of C1 protein kinase C homology domains. Protein Sci 6, 477–480. 9041654 10.1002/pro.5560060228 PMC2143645

[9] Kazanietz MG (2002) Novel “nonkinase” phorbol ester receptors: the C1 domain connection. Mol Pharmacol 61, 759–767. 11901214 10.1124/mol.61.4.759

[10] Nishizuka Y (1992) Intracellular signaling by hydrolysis of phospholipids and activation of protein kinase C. Science 258, 607–614. 1411571 10.1126/science.1411571

[11] Ron D and Kazanietz MG (1999) New insights into the regulation of protein kinase C and novel phorbol ester receptors. FASEB J 13, 1658–1676. 10506570

[12] Kim SC and Wang X (2020) Phosphatidic acid: an emerging versatile class of cellular mediators. Essays Biochem 64, 533–546. 32602549 10.1042/EBC20190089

[13] Sakane F , Hoshino F and Murakami C (2020) New era of diacylglycerol kinase, phosphatidic acid and phosphatidic acid‐binding protein. Int J Mol Sci 21, E6794. 10.3390/ijms21186794 PMC7555651 32947951

[14] Stace CL and Ktistakis NT (2006) Phosphatidic acid‐ and phosphatidylserine‐binding proteins. Biochim Biophys Acta 1761, 913–926. 16624617 10.1016/j.bbalip.2006.03.006

[15] Zegarlinska J , Piascik M , Sikorski AF and Czogalla A (2018) Phosphatidic acid – a simple phospholipid with multiple faces. Acta Biochim pol 65, 163–171. 29913482 10.18388/abp.2018_2592

[16] Zhukovsky MA , Filograna A , Luini A , Corda D and Valente C (2019) Phosphatidic acid in membrane rearrangements. FEBS Lett 593, 2428–2451. 31365767 10.1002/1873-3468.13563

[17] Sakane F , Imai S , Kai M , Yasuda S and Kanoh H (2008) Diacylglycerol kinases as emerging potential drug targets for a variety of diseases. Curr Drug Targets 9, 626–640. 18691010 10.2174/138945008785132394

[18] Sakane F , Mizuno S and Komenoi S (2016) Diacylglycerol kinases as emerging potential drug targets for a variety of diseases: an update. Front Cell Dev Biol 4, 82. 27583247 10.3389/fcell.2016.00082 PMC4987324

[19] Sakane F , Imai S , Kai M , Wada I and Kanoh H (1996) Molecular cloning of a novel diacylglycerol kinase isozyme with a pleckstrin homology domain and a C‐terminal tail similar to those of the EPH family of protein tyrosine kinase. J Biol Chem 271, 8394–8401. 8626538 10.1074/jbc.271.14.8394

[20] Crotty T , Cai J , Sakane F , Taketomi A , Prescott SM and Topham MK (2006) Diacylglycerol kinase δ regulates protein kinase C and epidermal growth factor receptor signaling. Proc Natl Acad Sci USA 103, 15485–15490. 17021016 10.1073/pnas.0604104103 PMC1622849

[21] Chibalin AV , Leng Y , Vieira E , Krook A , Bjornholm M , Long YC , Kotova O , Zhong Z , Sakane F , Steiler T *et al*. (2008) Downregulation of diacylglycerol kinase delta contributes to hyperglycemia‐induced insulin resistance. Cell 132, 375–386. 18267070 10.1016/j.cell.2007.12.035

[22] Miele C , Paturzo F , Teperino R , Sakane F , Fiory F , Oriente F , Ungaro P , Valentino R , Beguinot F and Formisano P (2007) Glucose regulates diacylglycerol intracellular levels and protein kinase C activity by modulating diacylglycerol‐kinase subcellular localization. J Biol Chem 282, 31835–31843. 17675299 10.1074/jbc.M702481200

[23] Sakai H , Kado S , Taketomi A and Sakane F (2014) Diacylglycerol kinase δ phosphorylates phosphatidylcholine‐specific phospholipase C‐dependent, palmitic acid‐containing diacylglycerol species in response to high glucose levels. J Biol Chem 289, 26607–26617. 25112873 10.1074/jbc.M114.590950 PMC4176214

[24] Lu Q , Komenoi S , Usuki T , Takahashi D and Sakane F (2018) Abnormalities of the serotonergic system in diacylglycerol kinase delta‐deficient mouse brain. Biochem Biophys Res Commun 497, 1031–1037. 29486157 10.1016/j.bbrc.2018.02.165

[25] Lu Q , Murakami C , Hoshino F , Murakami Y and Sakane F (2020) Diacylglycerol kinase delta destabilizes serotonin transporter protein through the ubiquitin‐proteasome system. Biochim Biophys Acta Mol Cell Biol Lipids 1865, 158608. 31891772 10.1016/j.bbalip.2019.158608

[26] Lu Q , Murakami C , Murakami Y , Hoshino F , Asami M , Usuki T , Sakai H and Sakane F (2020) 1‐Stearoyl‐2‐docosahexaenoyl‐phosphatidic acid interacts with and activates Praja‐1, the E3 ubiquitin ligase acting on the serotonin transporter in the brain. FEBS Lett 594, 1787–1796. 32134507 10.1002/1873-3468.13765

[27] Usuki T , Takato T , Lu Q , Sakai H , Bando K , Kiyonari H and Sakane F (2016) Behavioral and pharmacological phenotypes of brain‐specific diacylglycerol kinase delta‐knockout mice. Brain Res 1648, 193–201. 27423518 10.1016/j.brainres.2016.07.017

[28] Bunting M , Tang W , Zimmerman GA , McIntyre TM and Prescott SM (1996) Molecular cloning and characterization of a novel human diacylglycerol kinase ζ. J Biol Chem 271, 10230–10236. 8626588

[29] Goto K and Kondo H (1996) A 104‐kDa diacylglycerol kinase containing ankyrin‐like repeats localizes in the cell nucleus. Proc Natl Acad Sci USA 93, 11196–11201. 8855332 10.1073/pnas.93.20.11196 PMC38307

[30] Topham MK , Bunting M , Zimmerman GA , McIntyre TM , Blackshear PJ and Prescott SM (1998) Protein kinase C regulates the nuclear localization of diacylglycerol kinase‐zeta. Nature 394, 697–700. 9716136 10.1038/29337

[31] Baldanzi G , Ragnoli B and Malerba M (2020) Potential role of diacylglycerol kinases in immune‐mediated diseases. Clin Sci (Lond) 134, 1637–1658. 32608491 10.1042/CS20200389

[32] Merida I , Andrada E , Gharbi SI and Avila‐Flores A (2015) Redundant and specialized roles for diacylglycerol kinases alpha and zeta in the control of T cell functions. Sci Signal 8, re6. 25921290 10.1126/scisignal.aaa0974

[33] Liu Z , Chang GQ and Leibowitz SF (2001) Diacylglycerol kinase zeta in hypothalamus interacts with long form leptin receptor. Relation to dietary fat and body weight regulation. J Biol Chem 276, 5900–5907. 11078732 10.1074/jbc.M007311200

[34] Yakubchyk Y , Abramovici H , Maillet JC , Daher E , Obagi C , Parks RJ , Topham MK and Gee SH (2005) Regulation of neurite outgrowth in N1E‐115 cells through PDZ‐mediated recruitment of diacylglycerol kinase zeta. Mol Cell Biol 25, 7289–7302. 16055737 10.1128/MCB.25.16.7289-7302.2005 PMC1190239

[35] Mizuno S , Kado S , Goto K , Takahashi D and Sakane F (2016) Diacylglycerol kinase ζ generates dipalmitoyl‐phosphatidic acid species during neuroblastoma cell differentiation. Biochem Biophys Rep 8, 352–359. 28955976 10.1016/j.bbrep.2016.10.004 PMC5614480

[36] Taniguchi M and Okazaki T (2014) The role of sphingomyelin and sphingomyelin synthases in cell death, proliferation and migration‐from cell and animal models to human disorders. Biochim Biophys Acta 1841, 692–703. 24355909 10.1016/j.bbalip.2013.12.003

[37] Taniguchi M and Okazaki T (2020) Ceramide/sphingomyelin rheostat regulated by sphingomyelin synthases and chronic diseases in murine models. J Lipid Atheroscler 9, 380–405. 33024732 10.12997/jla.2020.9.3.380 PMC7521967

[38] Huitema K , van den Dikkenberg J , Brouwers JF and Holthuis JC (2004) Identification of a family of animal sphingomyelin synthases. EMBO J 23, 33–44. 14685263 10.1038/sj.emboj.7600034 PMC1271672

[39] Yamaoka S , Miyaji M , Kitano T , Umehara H and Okazaki T (2004) Expression cloning of a human cDNA restoring sphingomyelin synthesis and cell growth in sphingomyelin synthase‐defective lymphoid cells. J Biol Chem 279, 18688–18693. 14976195 10.1074/jbc.M401205200

[40] Vacaru AM , Tafesse FG , Ternes P , Kondylis V , Hermansson M , Brouwers JF , Somerharju P , Rabouille C and Holthuis JC (2009) Sphingomyelin synthase‐related protein SMSr controls ceramide homeostasis in the ER. J Cell Biol 185, 1013–1027. 19506037 10.1083/jcb.200903152 PMC2711605

[41] Muhle C , Bilbao Canalejas RD and Kornhuber J (2019) Sphingomyelin synthases in neuropsychiatric health and disease. Neurosignals 27, 54–76. 31877246 10.33594/000000200

[42] Murakami C , Hoshino F , Sakai H , Hayashi Y , Yamashita A and Sakane F (2020) Diacylglycerol kinase delta and sphingomyelin synthase‐related protein functionally interact via their sterile alpha motif domains. J Biol Chem 295, 2932–2947. 31980461 10.1074/jbc.RA119.012369 PMC7062193

[43] Murakami C and Sakane F (2021) Sphingomyelin synthase‐related protein generates diacylglycerol via the hydrolysis of glycerophospholipids in the absence of ceramide. J Biol Chem 296, 100454. 33621517 10.1016/j.jbc.2021.100454 PMC7988496

[44] Sato M , Liu K , Sasaki S , Kunii N , Sakai H , Mizuno H , Saga H and Sakane F (2013) Evaluations of the selectivities of the diacylglycerol kinase inhibitors R59022 and R59949 among diacylglycerol kinase isozymes using a new non‐radioactive assay method. Pharmacology 92, 99–107. 23949095 10.1159/000351849

[45] Niwa H , Yamamura K and Miyazaki J (1991) Efficient selection for high‐expression transfectants with a novel eukaryotic vector. Gene 108, 193–199. 1660837 10.1016/0378-1119(91)90434-d

[46] Reed SE , Staley EM , Mayginnes JP , Pintel DJ and Tullis GE (2006) Transfection of mammalian cells using linear polyethylenimine is a simple and effective means of producing recombinant adeno‐associated virus vectors. J Virol Methods 138, 85–98. 16950522 10.1016/j.jviromet.2006.07.024

[47] Knight MJ , Joubert MK , Plotkowski ML , Kropat J , Gingery M , Sakane F , Merchant SS and Bowie JU (2010) Zinc binding drives sheet formation by the SAM domain of diacylglycerol kinase δ. Biochemistry 49, 9667–9676. 20857926 10.1021/bi101261x PMC3035719

[48] Ding L , Bunting M , Topham MK , McIntyre TM , Zimmerman GA and Prescott SM (1997) Alternative splicing of the human diacylglycerol kinase ζ gene in muscle. Proc Natl Acad Sci USA 94, 5519–5524. 9159104 10.1073/pnas.94.11.5519 PMC20810

[49] Ogushi F , Ishitsuka R , Kobayashi T and Sugita Y (2012) Rapid flip‐flop motions of diacylglycerol and ceramide in phospholipid bilayers. Chem Phys Lett 522, 96–102.

[50] Uhlen M , Fagerberg L , Hallstrom BM , Lindskog C , Oksvold P , Mardinoglu A , Sivertsson A , Kampf C , Sjostedt E , Asplund A *et al*. (2015) Proteomics. Tissue‐based map of the human proteome. Science 347, 1260419. 25613900 10.1126/science.1260419

[51] Schindelin J , Arganda‐Carreras I , Frise E , Kaynig V , Longair M , Pietzsch T , Preibisch S , Rueden C , Saalfeld S , Schmid B *et al*. (2012) Fiji: an open‐source platform for biological‐image analysis. Nat Methods 9, 676–682. 22743772 10.1038/nmeth.2019 PMC3855844

