# Supplementary material for: Diacylglycerol kinase ζ interacts with sphingomyelin synthase 1 and sphingomyelin synthase‐related protein via different regions
Source: FEBS Open Bio. 2023 May 21;13(7):1333–45. doi: 10.1002/2211-5463.13628 (PMC10315710; doi:10.1002/2211-5463.13628)
Supplement: Supplementary file 1 — Fig. S1. Sequence alignment of SMSr‐SAMD and SMS1‐SAMD. (A) Sequence alignment of SMSr‐SAMD (aa 12–78) and SMS1‐SAMD (aa 7–70). Sequence alignment was created using Clustal Omega provided by EMBL's European Bioinformatics Institute (EMBL‐EBI). Compared with SMSr‐SAMD, white letters on a black background indicate fully conserved residues, and black letters on a gray background indicate strongly similar residues. (B) Amino acid identities between the SAMDs of SMSr and SMS1. Amino acid identity and similarity were determined using Pairwise Sequence Alignment provided by the European Molecular Biology Open Software Suite (EMBOSS). Fig. S2. Multiple sequence alignment of the C‐terminal regions of SMS1, SMS2, and SMSr. (A) Multiple sequence alignment of the C‐terminal regions of SMS1‐CT (aa 348–413), SMS2‐CT (aa 292–365), and SMSr‐CT (aa 364–415). Multiple sequence alignment was created using Clustal Omega provided by EMBL's European Bioinformatics Institute (EMBLEBI). Compared with SMS1‐CT, white letters on a black background indicate fully conserved residues, and black letters on a gray background indicate strongly similar residues. (B) Amino acid identities between the C‐terminal regions of SMS1, SMS2, and SMSr. Amino acid identity and similarity were determined using Pairwise Sequence Alignment provided by the European Molecular Biology Open Software Suite (EMBOSS). Fig. S3. Sequence alignment of DGKζ‐CD and DGKι‐CD. (A) Sequence alignment of DGKζ‐CD (aa 293–622) and DGKι‐CD (aa 374–702). Sequence alignment was created using Clustal Omega provided by EMBL's European Bioinformatics Institute (EMBL‐EBI). Compared with DGKζ‐CD, white letters on a black background indicate fully conserved residues, and black letters on a gray background indicate strongly similar residues. (B) Amino acid identities between DGKζ‐CD and DGKι‐CD. Amino acid identity and similarity were determined using Pairwise Sequence Alignment provided by the European Molecular Biology Open Software Suite [file FEB4-13-1333-s001.pdf]

Supplemental Materials

A

```
SMSr-SAMD  12- WTTKHVAVWLKDEGFFEYVDILCNKHRLDGITLLTLTEYDLRSPPLEIKVLGDIKRLMLSVRKLQKI
SMS1-SAMD   7- WSPKKVADWLLENAMPEYCEPLE---HFTGQDLINLTQEDFKKPPLCRVSSDNGQRLLDMIETLKME
```

B

	Amino acid identity	Amino acid similality
SMSr-SAMD vs SMS1-SAMD	30.9 %	48.5 %

Suppl. Fig. S1. Sequence alignment of SMSr-SAMD and SMS1-SAMD.
(**A**) Sequence alignment of SMSr-SAMD (aa 12–78) and SMS1-SAMD (aa 7–70). Sequence alignment was created using Clustal Omega provided by EMBL's European Bioinformatics Institute (EMBL-EBI). Compared with SMSr-SAMD, white letters on a black background indicate fully conserved residues, and black letters on a gray background indicate strongly similar residues. (**B**) Amino acid identities between the SAMDs of SMSr and SMS1. Amino acid identity and similarity were determined using Pairwise Sequence Alignment provided by the European Molecular Biology Open Software Suite (EMBOSS).

A

```
SMS1-CT  348- HTMANQQVLKEASQMNLLARVWWYRPFQYFEKNVQGIVPRSYHWPFPWPVVHLSRQV-KYSRLVNDT-------
SMS2-CT  296- HSMANEKNLKVSSQTNFLSRAWWFPIFYFFEKNVQGSIPCCFSWPLSWPPGCFKSSCKKYSRVQKIGEDNEKST
SMSr-CT  364- HTLANTRAYQQSR-----RARIWFPMFSFFECNVNGTVPNEYCWPFSKPAIMKRLIG-----------------
```

B

Amino acid identity	SMS2-CT	SMSr-CT
**SMS1-CT**	41.9 %	36.4 %
**SMS2-CT**		25.0 %

Amino acid similality	SMS2-CT	SMSr-CT
**SMS1-CT**	58.1 %	48.5 %
**SMS2-CT**		36.2 %

Suppl. Fig. S2. Multiple sequence alignment of the C-terminal regions of SMS1, SMS2, and SMSr.
(**A**) Multiple sequence alignment of the C-terminal regions of SMS1-CT (aa 348–413), SMS2-CT (aa 292–365) and SMSr-CT (aa 364–415). Multiple sequence alignment was created using Clustal Omega provided by EMBL’s European Bioinformatics Institute (EMBL-EBI). Compared with SMS1-CT, white letters on a black background indicate fully conserved residues, and black letters on a gray background indicate strongly similar residues. (**B**) Amino acid identities between the C-terminal regions of SMS1, SMS2, and SMSr. Amino acid identity and similarity were determined using Pairwise Sequence Alignment provided by the European Molecular Biology Open Software Suite (EMBOSS).

A

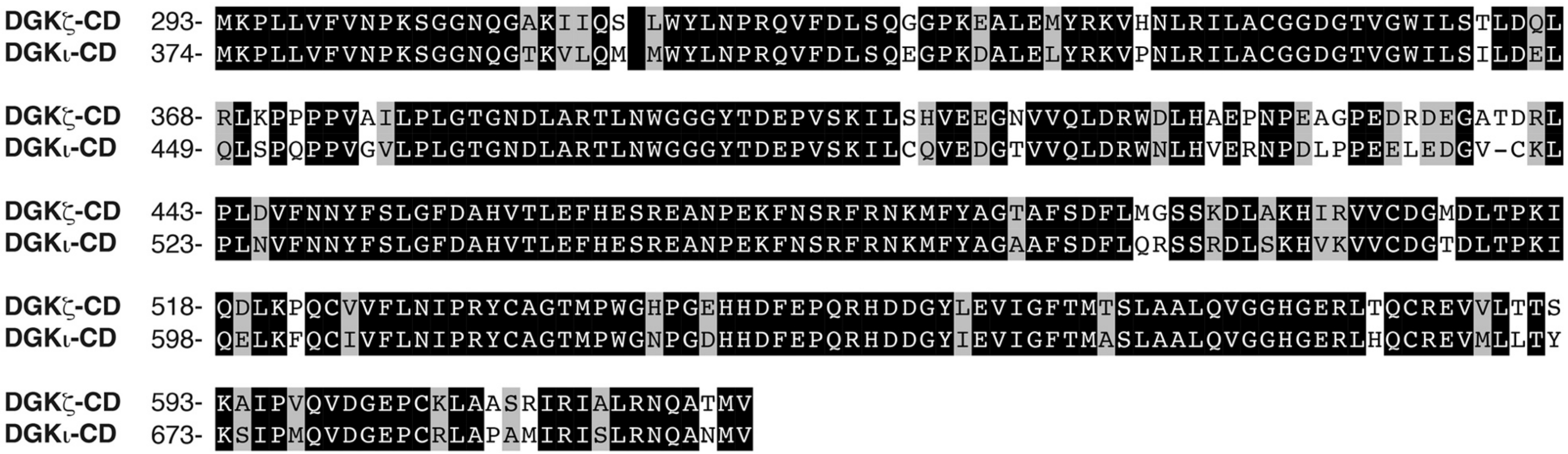

B

	Amino acid identity	Amino acid similality
DGKζ-CD vs DGKι-CD	81.2 %	90.6 %

Suppl. Fig. S3. Sequence alignment of DGKζ-CD and DGKι-CD.
(**A**) Sequence alignment of DGKζ-CD (aa 293–622) and DGKι-CD (aa 374–702). Sequence alignment was created using Clustal Omega provided by EMBL's European Bioinformatics Institute (EMBL-EBI). Compared with DGKζ-CD, white letters on a black background indicate fully conserved residues, and black letters on a gray background indicate strongly similar residues. (**B**) Amino acid identities between DGKζ-CD and DGKι-CD. Amino acid identity and similarity were determined using Pairwise Sequence Alignment provided by the European Molecular Biology Open Software Suite (EMBOSS).
